# Tumor-augmenting effects of gestational arsenic exposure on F1 and F2 in mice

**DOI:** 10.1186/s41021-016-0069-1

**Published:** 2017-03-01

**Authors:** Keiko Nohara, Takehiro Suzuki, Kazuyuki Okamura, Junya Matsushita, Shota Takumi

**Affiliations:** 1 Center for Health and Environmental Risk Research, National Institute for Environmental Studies, Tsukuba, 305-8506 Japan; 2 Graduate School of Pharmaceutical Science, Tokyo University of Science, Noda, 278-8510 Japan; 3 Department of Domestic Science, Kagoshima Women’s College, Kagoshima, 890-8565 Japan

**Keywords:** Arsenic, Gestational exposure, Hepatic tumor, F2, Multigenerational

## Abstract

The consequences of early-life exposure to chemicals in the environment are emerging concerns. Chronic exposure to naturally occurring inorganic arsenic has been known to cause various adverse health effects, including cancers, in humans. On the other hand, animal studies by Dr. M. Waalkes’ group reported that arsenite exposure of pregnant F0 females, only from gestational day 8 to 18, increased hepatic tumors in the F1 (arsenite-F1) males of C3H mice, whose males tend to develop spontaneous hepatic tumors later in life. Since this mice model illuminated novel unidentified consequences of arsenic exposure, we wished to further investigate the background mechanisms. In the same experimental model, we identified a variety of factors that were affected by gestational arsenic exposure, including epigenetic and genetic changes, as possible constituents of multiple steps of late-onset hepatic tumor augmentation in arsenite-F1 males. Furthermore, our study discovered that the F2 males born to arsenite-F1 males developed hepatic tumors at a significantly higher rate than the control F2 males. The results imply that the tumor augmenting effect is inherited by arsenite-F2 males through the sperm of arsenite-F1. In this article, we summarized our studies on the consequences of gestational arsenite exposure in F1 and F2 mice to discuss novel aspects of biological effects of gestational arsenic exposure.

## Background

Arsenic is widely distributed in the environment. Occupational exposure to various forms of arsenic compounds, as well as chronic exposure to naturally occurring background arsenic have been shown to cause serious health problems, including skin lesions, cardiovascular diseases, neuronal disorders, and cancers, in many areas in the world [1–6]. Arsenic is classified as a Group 1 human carcinogen by the International Agency for Research on Cancer (IARC). Numerous epidemiological studies have identified associations between chronic background inorganic arsenic exposure from drinking water with the risk of cancers in multiple organs, such as skin, lung, liver, bladder and kidney [1, 5].

The major arsenic species of concern in drinking water are inorganic arsenite (trivalent) and arsenate (pentavalent). They affect biological processes and cause toxic effects through a number of modes of actions (MOA), such as interacting with biological components in the sulfhydryl group, producing oxidative stress, and altering signal transduction [1, 3]. Epigenetic alterations such as DNA methylation changes are also implicated in the toxicity and carcinogenicity of arsenic [7–10]. Previous studies in bacteria and mammalian cells in vitro reported that arsenic has no or weak mutagenicity [1, 5]. On the other hand, since inorganic arsenite and arsenate have been shown to be metabolized into organic arsenic compounds having higher toxicity [11], we investigated mutagenicity of inorganic arsenite in transgenic mice developed for detecting in vivo mutations, as described later [12]. The results showed that arsenic exposure prominently increases G:C to T:A transversion, which is induced by oxidative stress. Thus, the involvement of mutation in arsenic-induced cancer augmentation needs to be reconsidered.

One of the emerging concerns about chemical exposure is the consequences of early-life exposure. The adverse effects of gestational exposure to environmental factors can lead to adult-onset diseases in the offspring and also in subsequent generations [13–15]. Epidemiological studies reported an association between arsenic exposure *in utero* as well as early childhood and an elevated risk of cancers [4, 16, 17]. In a mouse model, Waalkes and colleagues reported that gestational arsenite exposure of C3H mice, whose males are predisposed to spontaneously develop hepatic tumors in adulthood [18, 19], increases the hepatic tumor incidence in their F1 male offspring [20, 21]. In the same mouse model, we recently showed that gestational arsenite exposure of C3H mice increases hepatic tumor incidence not only in the males of F1 (arsenite-F1), but also in the males of the offspring of arsenite-F1 (arsenite-F2) [22, 23].

In this article, we will start with a brief overview of animal models for studying carcinogenicity of arsenic in the literature. Then, we summarize our studies on the cancer augmenting effects of gestational arsenite exposure in arsenite-F1 and arsenite-F2 males and discuss their mechanisms, including epigenetics and mutation.

## Animal models detecting arsenic carcinogenesis

Although animal studies to test arsenic carcinogenicity by dosing inorganic arsenic have a long history from the early 1900s, only a limited number of positive results have been observed [1, 5]. Convincing results were obtained in the 1990s when cancer promoting effects of the main metabolite of inorganic arsenic, dimethylarsinic acid (DMA^V^), given at 50-400 ppm in drinking water for 24 weeks, were investigated in rats pretreated with carcinogens [24]. Significant tumor promoting effects of DMA^V^ in drinking water was also reported by exposure at 10-100 ppm for 32 weeks in another tumor model in rats [25] and at 400 ppm for 25 weeks in a mice model [26]. Following studies showed that administering only DMA^V^ in drinking water induced bladder tumors in F344 rats at 50 and 200 ppm for 2 years [27], and augmented lung tumors in A/J mice, which spontaneously develop lung tumors, at 400 ppm for 50 weeks [28].

Arsenic concentrations in groundwater are generally lower than 10 ppb. On the other hand, epidemiological studies have reported higher levels of arsenic in drinking water in some endemic areas where the association of cancer risk and arsenic exposure by drinking water have been detected. The average levels of arsenic in most of those endemic areas were at most up to 1 ppm [5]. Thus, the results of animal studies suggested that rodents are much more refractory to the carcinogenicity of arsenic compared to humans. Given that animal models are pivotal to elucidate the molecular mechanisms of carcinogenicity, key factors which produce the difference in susceptibility to arsenic carcinogenicity between humans and rodents should be addressed to better understand the mechanism and estimate its risk.

In 2003, more evidence on the tumor augmenting effects of arsenic was reported by Waalkes et al. [20]. They demonstrated that administration of pregnant C3H mice with drinking water containing 42.5 or 85 ppm sodium arsenite only from day 8 to 18 of gestation significantly augmented hepatic tumor incidence in the male offspring at 74 weeks of age. The study showed that fetuses are highly susceptible to the tumor promoting effects of arsenic compared to adult animals. Subsequently, Waalkes and colleagues reported hypomethylation of the promoter region of the estrogen receptor α (*ERα*) and upregulation of *ERα* expression in the normal tissue of tumor-bearing livers in the F1 males gestationally exposed to arsenic, compared to the normal tissues of control mice [21]. Their study showed the possibility that epigenetic regulation of *ERα* expression is implicated in cancer augmentation in the exposed F1 males, since estrogen augments cellular growth. These results encouraged us to further investigate the mechanisms of the tumor augmenting effects of arsenic, as described below.

## Tumor augmenting effects in the liver of offspring by maternal arsenic exposure

The accumulated body of previous studies reported that arsenic acts as a tumor promoter through genetic, epigenetic, and metabolic alterations [25, 29, 30]. For exploring the mechanism of the augmenting effects of gestational arsenic exposure on the complex tumorigenic process, we investigated several factors, such as gene expression, mutation, and epigenetics in the C3H mice model established by Waalkes et al. [20]. As described below, we identified multiple factors that may be involved in the hepatic tumor augmentation around 74 weeks of age in arsenite-F1 [22].

## Late-onset gene expression changes, metabolic change and oxidative stress

**Fig. 1:**
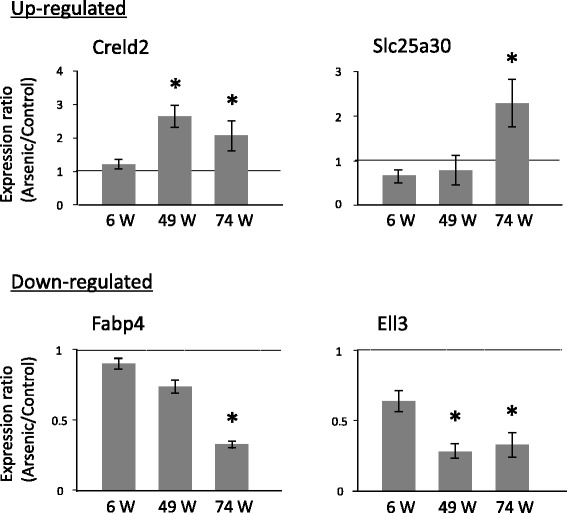
Late-onset changes in gene expression in the non-tumor-bearing livers of F1 male mice gestationally exposed to arsenic. Expression of four genes in the livers of control and gestationally arsenic-exposed mice was measured by real-time PCR at 6, 49, and 74 weeks of age and normalized to the expression of cyclophilin B (CPB). The graphs show the ratio of expression in the arsenic group normalized to expression in the control group. The data shown are the means ± S.E. (*n* = 11 for 6 w, *n* = 4 for 49 w, *n* = 8 for 74 w). * significant difference between the two groups at *p* < 0.05 (21)

Global gene expression analysis of as yet non-tumor-bearing livers at 74 weeks of age showed that two genes (*Creld2* and *Slc25a30*) are significantly upregulated and two genes (*Fabp4* and *Ell3*) were significantly downregulated more than 2-fold in arsenite-F1 males compared to the control males. Interestingly, time course analyses at 6, 49 and 74 weeks of age showed that the expression changes of the 4 genes are late onset events, since their expressions were not different in the control and arsenite-F1 males at 6 weeks of age and the difference between the two groups were firstly detected at 49 or 74 weeks of age (Fig. 1) [22].

The upregulation of *Creld2* and downregulation of *Fabp4* suggested the involvement of lipid metabolism, since *Creld2* is an endoplasmic reticulum (ER) stress-inducible gene [31] and lipogenesis is activated by ER stress [32]. Fabp4 is one of the fatty acid–binding proteins and is known to play a role in effluxing fatty acids from adipocytes [33]. Lipid accumulation in the liver can lead to the promotion of tumorigenesis thorough oxidative stress production [34]. The measurement of lipid contents in the normal livers at 74 weeks of age showed a 16% increase of triglyceride in the arsenite-F1 males in comparison with the control males. We also detected the suppression of *glycerol-3-phosphate acyltransferase-1* (*Gpat-1*), one of the target genes of sterol regulatory element-binding protein 1 (*Srebp1*), the central transcription factor which regulates the levels of cholesterol and fatty acids [35]. We also detected upregulation of a representative oxidative stress inducible gene, *HO-1*, in the normal livers of arsenite-F1 males compared to the control males. These results suggest that gestational arsenic exposure induces late-onset changes in gene expression leading to changes in lipid metabolism and augmentation of oxidative stress, which promote tumorigenesis [22]. Further biochemical studies are necessary to confirm the levels of involved factors such as the enzymes.

## Epigenetic changes

Epigenetic mechanisms, such as genomic DNA methylation, histone modification and small RNA manipulation, play a role in the regulation of gene expression and thereby adjust biological functions or cause disorders [36–38].

DNA methylation occurring primarily at the 5-carbon of the cytosine in CpG dinucleotides is produced by DNA methyltransferases (DNMTs) and the level is regulated by the balance between DNA methylation and active and/or passive DNA demethylation [36, 38]. Global DNA hypomethylation, i.e., a reduction in the total amount of 5-methylcytosine (5meC), is a well-known feature of cancer cells and leads to genomic instability, whereas hypomethylation and hypermethylation of DNA in promoter regions can facilitate and suppress gene expression, respectively. Post-translational modifications, such as methylation and acetylation, of histone tails are also involved in the regulation and maintenance of transcription levels [36].

We investigated the involvement of epigenetic regulation for the four genes (*Creld2*, *Slc25a30, Fabp4* and *Ell3*) whose expressions were different between the normal livers of the control and arsenite-F1 males [22]. As a result, a clear association was observed between the suppression of *Fabp4* expression in arsenite-F1 males and the significant increase in H3K9me2, which is the hallmark of heterochromatin where transcription is suppressed [36]. On the other hand, no change of DNA methylation status in the promoter region was observed in any genes.

To extend our knowledge on DNA methylation changes by gestational arsenic exposure, we performed genome wide DNA methylation analysis of normal hepatic tissues and hepatic tumor tissues in the control and arsenite-F1 males by the methylated DNA immunoprecipitation (MeDIP)-CpG island microarray method [39]. We detected 16 DNA regions where methylation statuses were altered in the tumor tissues of arsenite-F1 males compared to the normal livers of the control F1 mice. Among them, we found that a gene body region of *Fosb*, a member of the oncogene family, is hypermethylated in the tumors of arsenite-F1 compared to the tumors of the control males and the gene expression was significantly increased corresponding to the DNA methylation level. Several studies reported that higher DNA methylation in the gene body region upregulates gene expression [40–42]. Hence, the results of our study suggest that gestational arsenic exposure affects gene expression by inducing DNA methylation in the gene body region of *Fosb.* Although the role of *Fosb* in carcinogenesis is still unknown, gene expression change of *Fosb* may play a part in tumor augmentation in arsenite-F1 males.

## Ha*-ras* mutation

Several types of cancers in humans and mice frequently contain mutation of the *ras* oncogene family (Ha-, Ki-, and N-*ras*), which keeps the protein in the active form and is thought to be an early event occurring in the initiation stage and in driving tumorigenesis in various processes [19, 43]. Previous studies reported that 9–60% of the spontaneous hepatic tumors of C3H mice harbor a Ha*-ras* mutation, primarily at codon 61 [17].

In our study, we detected three types of Ha-*ras* mutations at codon 61 (C61A, A61T and A61G), with C61A mutations predominating, in the hepatic tumor tissues of the control and arsenite-F1 males around 74 weeks of age. These mutations were shown to be somatically acquired, since no mutations were detected in Ha-*ras* in the normal tissues of the tumor-bearing livers. We further found that gestational arsenite exposure tended to increase the percentage of livers having tumors containing the Ha-*ras* mutation, and the percentage of livers with C61A Ha-*ras* mutation was more than doubled in arsenite-F1 males compared to the control males [22]. C to A mutations, that is, G:C to T:A transversions, are induced following the formation of 8-hydroxy-2-deoxyguanosine (8-OHdG), a representative product of oxidative DNA damage [44]. As described above, our results suggested that gestational arsenic exposure increases oxidative stress in a late-onset manner through lipid metabolism alteration. Thus, such an increase in oxidative stress later in life may be involved in the increase in hepatic Ha-*ras* mutation and tumor augmentation.

On the other hand, previous studies performed in bacteria and mammalian cells in vitro have reported that inorganic arsenic shows no or weak mutagenicity. Inorganic arsenic is metabolized in vivo into organic compounds and their toxicities vary depending on the forms [11]. However, the mutagenicity of arsenic has not been fully assessed in animal models. Thus, using *gpt* delta transgenic mice, in which *gpt* gene cassettes are integrated on genomic DNA for detecting in vivo mutations [45], we investigated in vivo mutagenicity of orally exposed arsenite [12]. Male *gpt* delta mice were given drinking water containing 85 ppm sodium arsenite for 3 weeks, and mutations in the hepatic genome were assayed 2 weeks later. The assay showed approximately a 1.5-fold significant increase in average mutation frequency in the arsenite-treated mice in comparison with the control mice. DNA sequencing of the *gpt* gene showed a marked increase in G:C to T:A transversions (46% of all mutations) in the arsenite-treated mice compared to that in the control mice (5% of all mutations). We also detected a significant increase in 8-OHdG in the livers of arsenite-treated mice. These results demonstrated that arsenite has mutagenicity, particularly inducing an oxidative-stress-associated G:C to T:A transversions in vivo [12]. Gestational arsenic exposure may increase the chance of C to A mutation at codon 61 in Ha-*ras* in fetus hepatic cells via oxidative stress production and the mutation may be involved in tumorigenic transformation.

## Retrotransposon Long interspersed nuclear element-1 (Line-1) Activity

**Fig. 2:**
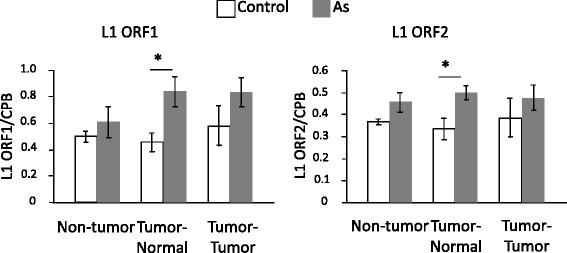
Increased Line-1 RNA expression in the livers of adult F1 male mice gestationally exposed to arsenic. Expression of *ORF1* and *ORF2* in normal adult livers, normal tissue from tumor-bearing livers, and tumor tissue from tumor-bearing livers were measured by real-time PCR and normalized to the expression of CPB. Results are reported as means ± S.E. (*n* = 6). * significant difference between the two groups at *p* < 0.05 (21)

Line-1 retrotransposon is a major class of transposable elements in humans and mice. Although most transposable elements have been rendered inactive, Line-1 is capable of autonomous retrotransposition, which exerts mutagenic consequences by inserting into the genome or by causing breaks in double-stranded DNA [46, 47]. Both ORF1 and ORF2 proteins, the products of open reading frames 1 and 2, are required for its retrotransposition. In our study, we detected a significantly higher expression of Line-1 ORF1 and ORF2 in the normal tissues of tumor-bearing livers of arsenite-F1 males in comparison with the control males at 74 weeks of age (Fig. 2), and found that it is induced in a late-onset manner since it was not detected at 6 or 49 weeks of age [22]. These results suggest that gestational arsenic exposure induces late-onset Line-1 activation in the liver and promotes genetic instability. Recent study in vitro also reported that arsenic exposure to HepG2 cells significantly increased Line-1 retrotransposition frequency [48].

**Fig. 3:**
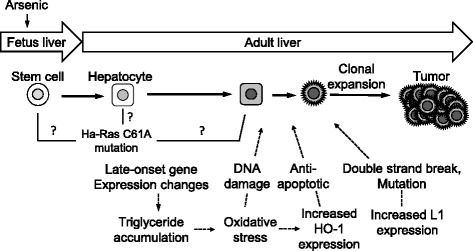
Possible actions of gestationally exposed arsenic in the hepatic tumorigenesis in F1 mice (22)

Taken together, our studies so far have proposed the involvement of multiple factors in the tumor-augmentation in the arsenite-F1 (Fig. 3).

## Germ cell-transmitted effects in the F2 generation

**Fig. 4:**
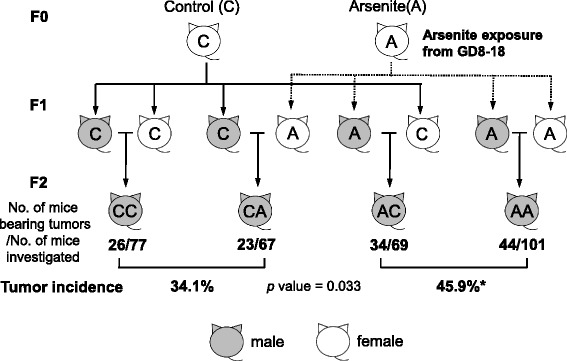
Increase in the tumor incidence in the F2 male offspring born to arsetite-F1 males but not to ansenite-F1 females. The F2 mice were macroscopically examined for hepatic tumors at 75-82 weeks of age in an age-matched manner (23). The difference between the tumor incidences in the two groups was analyzed by chi-square test

Our recent study further demonstrated that only gestational arsenic exposure in the Waalkes’ model increases tumor incidence even in the F2 generation (arsenite-F2) [23]. The routes through which gestational arsenic exposure affects F1 and F2 are totally different. The organs of F1 offspring are directly exposed to arsenic during the fetal period. Rapidly growing fetus organs are highly susceptible to chemicals since chemicals often disturb cellular differentiation and affect their plasticity [49]. Changes in hormone levels and metabolisms in the exposed mothers can also affect fetal condition. On the other hand, the F2 effect is transmitted through exposed germ cells of F1 to the F2 generation. To elucidate the transmission route of the F2 effect, we investigated whether the tumor augmenting effects of gestational arsenic exposure originate with either male or female, or both, of the F1 by reciprocal crossing between the control and arsenite-F1 males and females (Fig. 4) [23]. The results showed that the F2 males born to arsenite-F1 males (AC and AA, Fig. 4) developed tumors at a significantly higher rate than the F2 males born to the control F1 males, irrespective of exposure of F1 females (CC and CA, Fig. 4). These results showed that the sperm of F1 fathers is responsible for the transmission of tumor augmenting effects by gestational arsenite exposure into the F2 males.

Epigenetic modifications of the genome (epigenome), particularly DNA methylation modification, of germ cells play a pivotal role in development. The DNA methylation pattern is known to be dynamically reprogrammed in pre-implantation embryos as well as in primodial germ cells (PGCs), the precursor cells for both spermatozoa and oocytes, emerging in a fetus [50]. The epigenomes of these stages are thought to be vulnerable to chemical exposure [13–15]. In our study, pregnant mothers were exposed to arsenite in the gestational period from GD8, around which time DNA methylation of PGCs in the fetus undergoes tremendous reprogramming. Arsenite may disturb the epigenome of PGCs in the F1 males and augment hepatic tumors in the F2 males. At present, we do not know what changes cause tumor augmentation in the F2 livers. To clarify this, identification of tumor augmenting factors in the F2 livers and epigenetic alterations in the sperm, which leads to tumor augmentation in the F2, by gestational arsenite exposure is required.

Furthermore, gestational exposures to a variety of environmental factors, including several chemicals, have been reported to cause not only multigenerational (in F1 and F2), but also transgenerational effects that are inherited by the F3 and following generations [14, 15]. In addition to the tumor augmenting effects, we recently demonstrated that arsenic exposure of pregnant mice causes behavioral inflexibility and impaired cortical structure in the F1 offspring [51]. A recent study by another group reported that arsenic exposure of pregnant mice leads to obesity and early onset of the vaginal opening in the F1 females [52]. The consequences of gestational exposure to arsenic also needs to be addressed multigenerationally and transgenerationally from a long-term perspective.

## Conclusion

Our studies showed that gestational arsenite exposure induces late-onset gene expression changes in normal livers and expansion of tumors having epigenetic and genetic changes in the F1 males of C3H mice. Genomic instability shown by up-regulation of Line-1 expression and metabolic changes were implicated in hepatic tumor augmentation. Furthermore, our study demonstrated that tumor augmenting effects by gestational arsenite exposure were transmitted to the F2 males through the F1 sperm. Animal studies are indispensable for elucidating molecular mechanisms and predicting unclarified effects of chemical exposure. Since rodents are demonstrated to be highly refractory to arsenic compared to humans, experimental models using mice and rats adopt higher doses of arsenic to develop symptoms similar to those which are observed in humans. Considering the differences between rodents and humans, further studies on the mechanisms are necessary to provide important data to estimate multigenerational tumor-augmenting effects of arsenic.

## Abbreviations

Arsenite-F1: The offspring of mother gestationally exposed to arnenite
Arsenite-F2: The offspring of arsenite-F1
CPB: Cyclophilin B
DMA^V^: Dimethylarsinic acid
ER: Endoplasmic reticulam
ERα: Estrogen receptor α
Line-1: Long interspersed nuclear element-1
PGC: Primodial germ cell
